# A Pilot Study on RTE Food Purchasing and Food-Related Behaviors of College Students in an Urbanized Area

**DOI:** 10.3390/ijerph19063322

**Published:** 2022-03-11

**Authors:** Jinkyung Choi

**Affiliations:** Department of Food Science and Nutrition, Pukyong National University, Buasn 48513, Korea; choijk@pknu.ac.kr

**Keywords:** ready to eat food, college students, nutrition, food behaviors

## Abstract

Ready-to-eat (RTE) food is widely used, and younger age groups are increasingly purchasing these items. This study investigated college students’ consumption of RTE foods and food-related behaviors such as dietary habits, lifestyle, eating out behaviors, and demographic characteristics. We used quantitative methods to gather data. A questionnaire was developed from previous studies and was self-administered to college students in Daejeon, Republic of Korea. Descriptive analysis, ANOVA, and Chi-square tests were conducted to investigate RTE food consumption behaviors and food-related lifestyle factors. A total of 285 data points was entered for analysis. This study found that BMI and gender significantly impacted dietary habits. Residence type and gender showed significant effects on dining out partners. Furthermore, results showed the time of snack consumption and RTE food were similar, suggesting that a snack was replacing college students’ meals or that they might consider RTE food as a snack, or vice versa. A nutrition intervention program for college students should be implemented to encourage a healthy diet.

## 1. Introduction

Changes in family structure and socio-cultural factors have led to new ideas regarding meals. Previously, having a meal was considered eating warm food with family or friends sitting at a table. However, now it is not unusual to see a single consumer eating at a restaurant or having a meal at a convenience store. Having a meal has become a burden for some young consumers and single-person households, which has led them to prefer food that is easy to cook and prepare. The ready-to-eat (RTE) food market has sharply increased to about 500 billion won in 2017 from approximately 78 billion won in 2013. This is mainly due to an increase in single-person households and the economic downturn [1]. Single-person households and young consumers are the primary consumers of food prepared away from home. RTE food in convenience stores is mostly consumed by individuals in their teens or 20 s [2], suggesting college students are major consumers of RTE food.

College students have unique food consumption patterns. They often prefer food that is quick and easy [3], select food that is convenient to prepare [4], and food that tastes good [5]. They also prefer convenient food and fast food [6,7,8]. It was reported that college students are well-known for their inappropriate food consumption habits [9]. They have low consumption of fruits, vegetables, and whole grains [10,11]. In Korea, college students have irregular eating habits, such as skipping meals, snacking, drinking alcohol to excess, and they prefer snacks as meals but are concerned with nutritional value [12]. For example, they drink water, eat vegetables, consume protein, such as fish, and avoid salty food [12].

The eating environment of college students affected their eating habits [13,14]. The eating environment of college students allows for easy weight gain [15] and promotes an unhealthy lifestyle [14,16]. Skelton explored college students’ perceptions of the environmental issues toward healthy eating on campus and found limited access to healthy food due to physical market locations and time [17]. Moreover, many vending machines provide “junk” food, which is tempting [17]. Many convenience stores near colleges in Korea sell ready-to-eat food. In addition, the price of healthy food may prohibit college students’ consumption of vegetables and fruits [14,17]. Housing type significantly impacts the food behaviors of college students [14].

In most Asian countries, demographic characteristics had a less significant impact on the purchasing of RTE products. However, in general, people seemed to have a positive opinion towards RTE product outlets [18]. Consumers believe that RTE products can save food preparation time [19]. Convenience was the major reason for consumers gave for why they repurchase RTE products [20]. Satisfaction with RTE did not have a significant relationship with dietary style [21,22].

In Japan, convenient stores appeal to young consumers as enhancing their well-being [23], which may mean ‘convenience’ of product is not a stand-alone value but must support health. Interest in health has been increasing, as has the obesity rate [24]. The obesity rate of those aged 20–39 was 29% [25], while the rates of overweight and obesity are 36.7% [26]. The types of convenient foods are home meal replacement (HMR) products, fast food, and ready-to-eat food mostly consumed by college students as meals [11,27]. RTE food sold at convenience stores is cooked mainly via stir-frying and shows caloric content which is over for recommended caloric contents for women [28].

Therefore, this study researched the relationships among food behaviors, obesity, and health by examining college students’ RTE food purchase behaviors. The study investigated dietary habits and lifestyle factors such as exercise, sleep, and dietary supplement use of college students. In addition, we examined characteristics of college students, including demographic data and eating out and RTE food behaviors.

## 2. Methods

### 2.1. Study Procedure and Participant Information

In this study, a quantitative method was used to collect data. College students in Daejeon, Republic of Korea, were recruited as study participants. They were informed about the purpose of the study and asked to participate in a 3-week on-campus survey. Their participation agreement was also obtained. Our target sample size was 150, which was determined with an F-test via the G*Power program. For our t-test, a sample size of 111 was needed. After the two sample sizes and the response rate were considered, a total of 310 questionnaires were distributed, and 289 were returned. Then, four unusable responses such as incompletion were removed, so 285 surveys were used in the final analysis. This study was approved by the institutional review board (1041549-190709-SB-77).

### 2.2. Measurements

A questionnaire was developed based on previous studies [29,30,31]. The questionnaire surveyed dietary habits, eating out and RTE food consumption behaviors, and demographic characteristics. We asked six questions regarding dietary habits, such as if they consider ‘three major nutrients’, ‘nutritional value of food’, ‘calories of food’, ‘avoiding salt and sugar’, ‘appropriate portions of food’, and ‘sanitation’ important. In addition, eating out behaviors were assessed by questions such as ‘with whom do you dine out?’ ‘what food do you prefer when dining out?’ and ‘what is your budget for dining out?’ RTE food consumption behaviors were asked, such as ‘what is the purpose of purchasing RTE food?’, ‘what is your ethnic preference for RTE food?’, ‘what is your budget for RTE food?’, ‘what do you consider when purchasing RTE food?’, and ‘do you make nutritional considerations when purchasing RTE food?’ Lastly, we questioned respondents on their RTE food purchase time.

### 2.3. Statistical Analysis

The gathered data were coded using Excel and analyzed using SPSS Win (version 24.0. SPSS Inc, Chicago, IL, USA). Descriptive analyses were used on respondents’ dietary styles, dining out and RTE food consumption behaviors, and demographic characteristics. An independent t-test and ANOVA with Scheffe’s test were run to find the significances between dietary habits and BMI, types of residence, grade, and gender. Chi-square was used to investigate the significance of eating out behaviors and demographic characteristics. In addition, the relationship between RTE consumption behaviors and demographic characteristics was investigated using Chi-squares.

## 3. Results

### 3.1. Respondent Characteristics

Table 1 shows the characteristics of the respondents. Respondents included men (44.6%) and women (55.4%), and most were single (98.34%). Respondents included freshmen (38.9%), juniors (35.8%), sophomores (15.1%), and seniors (10.2%). Most lived alone (56.8%), followed by those living with their parents (33.2%), living in a dormitory (8.7%), or living in a room and board situation (1.2%). Most respondents were normal weight (57.6%), followed by overweight (17.9%), obese (16.6%), and underweight (7.9%). The majority of the respondents were non-smokers (78.9%) and not vegetarians (98.8%).

### 3.2. Dietary Habits of the Respondents

A set of six questions measured respondents’ beliefs regarding three major nutrients, the nutritional value of food, calories in food, avoidance of salt and sugar, food portions, and sanitation. Respondents frequently considered three major nutrients (41.6%), followed by sometimes (29.1%), almost always (24.2%), and rarely (5.2%). Respondents considered their diet’s nutritional value sometimes (43.7%), frequently (25.7%), rarely (24.2%), and almost always (6.4%).

Respondents reported that they consider food calories rarely (34.5%), sometimes (32.6%), frequently (24.6%), and almost always (8.3%). Respondents considered sugar and salt rarely (44.6%), sometimes (42.5%), frequently (9.2%), and almost always (3.7%). Participants reported that they considered appropriate portion sizes frequently (41.1%), sometimes (35.6%), almost always (15.3%), and rarely (8.0%). Regarding sanitation, respondents were concerned frequently (44.8%), almost always (35.6%), sometimes (16.3%), and rarely (3.4%).

ANOVA showed that respondents’ BMIs were significantly different based on their consideration of when they eat three major nutrients (M = 2.85, SD = 0.848, F = 3.388, *p* < 0.05); the nutritional value of food (M = 2.12, SD = 0.858, F = 3.447, *p* < 0.05); and food calories (M = 2.07, SD = 0.960, F = 5.207, *p* < 0.01) (Table 2). Overweight respondents (M = 3.02, SD = 0.758) were more concerned with the three major nutrients than those that were underweight (M = 2.48, SD = 0.847, *p* < 0.05). Those of a normal weight were more concerned about the nutritional value of food (M = 2.18, SD = 0.884) than those that were underweight (M = 1.73, SD = 0.640, *p* < 0.05). Regarding food calories, underweight individuals (M = 1.50, SD = 0.679) were less concerned than those of a normal weight (M = 2.09, SD = 0.941, *p* < 0.05) or obese individual (M = 2.20, SD = 0.901, *p* < 0.05). Grade did not have a significant impact on these eating characteristics, but gender showed a significant impact on the nutritional value of food (t = 5.079, *p* < 0.001) and avoidance of salt and sugar (t = 2.354, *p* < 0.5). Men showed a greater consideration of the nutritional value of food (M = 2.41, SD = 0.917) than women (M = 1.93, SD = 0.748) and were more careful to avoid salt and sugar (M = 1.83, SD = 0.833) than women (M = 2.58, SD = 0.813).

### 3.3. Characteristics of Eating out Behaviors

Table 3 shows the characteristics of eating out behaviors. The majority of the respondents eat out with their friends (68.5%), family (21.7%), alone (8.3%), with others (1.3%), and with colleagues (0.3%). This showed a significant relationship with residence type (χ^2^ = 29.206, *p* < 0.01) and gender (χ^2^ = 9.918, *p* < 0.05).

Respondents’ preferred food when eating out was Korean (50.2%), Western (18.0%), Japanese (14.8%), others (11.7%), and Chinese (5.4%). The budget for eating out was 7000–8999 won (34.5%), 5000–6999 won (21.2%), 11,000–12,999 won (17.1%), 9000–10,999 won (17.1%), ≥15,000 won (6.5%), 13,000–14,999 won (2.8%), and under 5000 won (1.2%). No significant relationships were found between preferred food when eating out and BMI, place of residence, or gender.

Dining out budget per person was 7000–8999 won (34%), 5000–6999 won (21.2%), 9000–10,999 won (17.1%), 11,000–12,999 won (17.1%), ≥15,000 (6.5%), 13,000–14,999 won (2.8%), and <5000 won (1.2%). There were no significant differences between budget for dining out and BMI, place of residence, or gender.

### 3.4. RTE Food Purchasing Behaviors

Table 4 shows the characteristics of RTE food purchasing behaviors. The respondents’ purposes for purchasing RTE foods included lunch (38.6%), dinner (22.9%), snacks (20.1%), breakfast (9.7%), and late-night snacks (8.8%). There were no significant differences between the purpose of RTE food purchase and BMI, residence type, or gender. The preferred RTE food was Korean (52.7%), others (19.0%), Western (15.9%), Japanese (7.9%), and Chinese (4.4%). In addition, the preferred food style was significantly impacted by gender (χ^2^ = 10.261, *p* < 0.05). The RTE food budget was 4000–4999 won (38.6%), <4000 won (31.5%), 5000–5999 won (23.4%), 6000–6999 won (3.7%), and 7000–7999 won (2.5%). No significant differences were found between BMI, types of residence, and gender.

Important factors that influenced the choice of HMT products included taste (43.3%), price (26.0%), portion size (18.1%), nutritional value (5.1%), brand (3.1%), low-calorie content (2.5%), and origin of the food ingredients (1.1%). These results were similar to a previous study (Tam et al., 2017) in which college students considered ‘taste’ as the most important factor when selecting food. Nutrients considered included calories (38.5%), carbohydrates (23.5%), protein (18.4%), fat (10.8%), and cholesterol (8.8%). This contradicts the fact that the majority of respondents did not consider calories in their daily diets but did consider them when purchasing RTE food. They may think RTE food is for special occasions or do not consider them as part of their daily diet.

**Figure 1:**
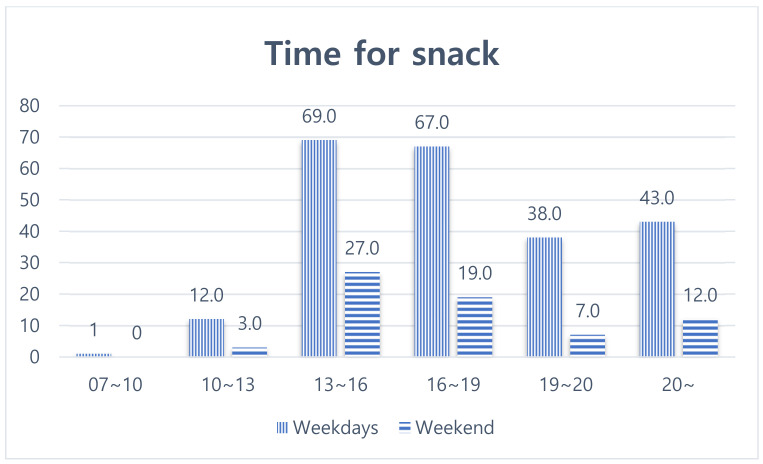
Time of snack during weekdays and weekends.

The purchase time of RTE food on weekdays was 13–16 (32.4%), 10–13 (19.1%), 19–20 (15.1%), after 20 (12.9%), and 7–10 (5.4%) (Figure 1). The purchase times of RTE food on the weekends were 13–16 (29.7%), 16–19 (25%), after 20 (18.8%), 19–20 (15.6%), 10–13 (9.4%), and 7–10 (1.6%), similar to weekdays.

**Figure 2:**
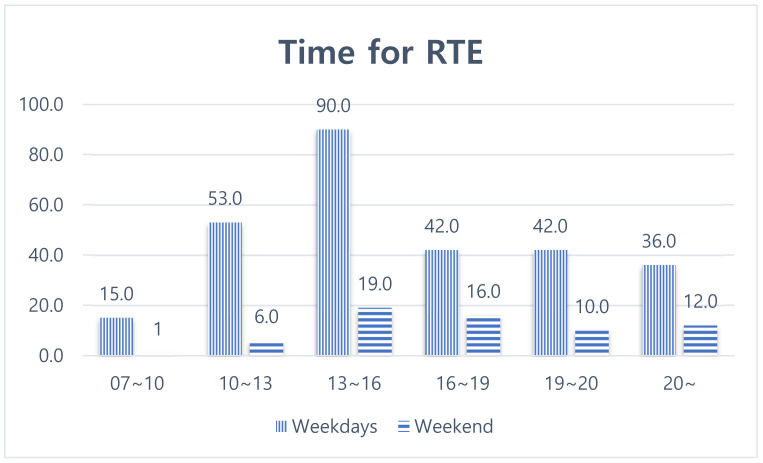
Time for RTE food during weekdays and weekends.

Timing of weekday snacks were 13–16 (30%), 16–19 (29.1%), over 20 (18.7%), 19–20 (16.5%), 10–13 (5.2%), and 7–10 (0.4%). Snack purchase times on weekends were 13–16 (39.7%), 16–19 (27.9%), over 20 (17.6%), 19–20 (10.3%), 10–13 (4.4%), and 7–10 (0.0%) (Figure 2). Snack purchase time was similar to RTE food purchase time, although there were some differences. RTE foods were purchased around lunch hours (1–4 p.m.) while respondents snacked at 1–4 p.m. and 4–7 p.m., indicating any time in the afternoon can be snack time.

## 4. Discussion

BMI results showed that the rates of overweight and obesity were similar to that of Western countries [26]. The Westernized diet can trigger obesity among college students. In addition, college students’ preferred food is often high in saturated fat and sodium, regardless of nationality. BMI was related to the consideration of three major nutrients, the nutritional value of food, and food calories. Interestingly, obese respondents demonstrated more concern regarding food calories than underweight and overweight individuals and for the three major nutrients than underweight individuals. The results suggested that respondents might misunderstand nutritional values. Male students were more concerned with the nutritional value of food and avoided salt and sugar, unlike the results of a previous study [32]. Females might consume more fruits and vegetables [3] and less high-energy foods [5]; however, men were more considerate of general nutritional value.

Residence type did not influence dietary habits. This differed from previous research [13,14] and might be why college students in the Republic of Korea have similar eating habits and lifestyles regardless of their residence type. Moreover, the majority of study respondents were living with their parents. Individual dietary habits may become similar to others’ habits due to social-cultural changes.

There were no differences in the purpose of RTE food based on BMI or residence type, which aligned with findings that there were no differences in dietary habits based on residence or gender. College students preferred RTE food for their lunch and consumed them for dinner or snacks between meals. Meal and snack time consumption patterns indicate that college students did not distinguish between meals and snacks when consuming RTE food. In other words, RTE food is consumed for meals and snacks, which suggests that the boundary between meals and snacks has become vague. Furthermore, the respondents’ late-night consumption of snacks and RTE foods suggests that college students must receive educational interventions on proper food consumption or daily dietary habits. Studies have demonstrated that their dietary habits differ from those of the general population [3], and they usually ignore healthy dietary behaviors, such as recommended food groups [33]. Given that the respondents were college students, they could enhance their understanding of healthy eating habits through on-campus classes or workshops [17].

There were some differences in the perception of dining out and consuming RTE food. College students might have considered that the cost of RTE foods was much lower than that of food dined out. This may be why dining out is perceived as an eating behavior with a different atmosphere compared to daily dietary behaviors. It is noteworthy that college students consider the taste and price of RTE food [5,12]. Calories were the most considered nutrients, followed by carbohydrates, protein, fat, and cholesterol. This suggests that college students perceive RTE foods as foods with high caloric content. Proper educational interventions are needed for college students. Moreover, eating environments affect dietary behaviors [14,17].

While individual efforts to maintain healthy eating are needed, RTE foods should be of high quality and meet nutritional guidelines. Most RTE food sold in convenient stores requires stir-frying or deep-frying cooking methods and has high sodium and fat content [28].

There are some limitations to this study. The survey was conducted in Daejeon, which is one of the largest cities in the Republic of Korea. Many students in this area either live with their parents or live alone, so they do not represent the general college student population. Hence, residence type might not affect several areas in the study. Furthermore, the small sample size and the high percentage of students living alone or at home might influence our results. For this reason, larger sample size and a larger student population in other areas might provide more significant data. In addition, this study did not measure satisfaction or repurchase intention, so further research is warranted to measure these factors. This study investigated the general dietary habits of college students in relation to RTE food. For future study, college students’ specific dietary behaviors (such as eating under stress, overeating, or skipping meals) should be investigated regarding RTE food consumption.

## 5. Conclusions

This research was conducted to investigate college students’ dietary habits, dining out behaviors, and RTE food consumption. Some findings of this study were similar to previous results, but other findings were still noteworthy. College students consume RTE foods for their lunch, dinner, and snacks, but they might underestimate the nutritional value of meals. Overweight and obese individuals considered nutritional value to a greater extent than underweight individuals did. Therefore, nutritional education among college students should be improved, and RTE foods should be prepared to meet nutritional guidelines.

This study indicated that college students considered eating RTE foods for daily consumption, not for special occasions, suggesting that RTE foods might no longer be appropriate for special occasions among all generations. Hence, further research should investigate consumers’ perception and purchasing behavior toward RTE food.

## Figures and Tables

**Table 1:** Characteristics of Demographics (n = 285).

Characteristics		Frequency	Valid Percentage (%)
Gender	Male	145	44.6
	Female	180	55.4
	Missing	2	
Marital status	Married	5	1.6
	Single	316	98.34
	Missing	6	
Grade	Freshman	126	38.9
	Sophomore	116	35.8
	Junior	49	15.1
	Senior	33	10.2
	Missing	3	
Place of residence	Live in board and lodging	4	1.2
	Live alone	183	56.8
	School dormitory	28	8.7
	Live with parents	107	33.2
	Missing	5	
	Below 18.5	23	7.9
BMI	18.5~22.9	167	57.6
	23~24.9	52	17.9
	25 and above	48	16.6
	Missing	37	
Do you smoke?	Yes	61	21.1
	No	228	78.9
	Missing	38	
Are you vegetarian?	Yes	4	1.2
	No	318	98.8
	Missing	5	

**Table 2:** Dietary habits of the respondents.

*I Usually Consider … when I Eat*		BMI	Grader	Gender
	Mean ± SD	F-Value		t-Value
3 major nutrients	2.85 ± 0.848	3.388 *	0.520	1.546
nutritional value of food	2.12 ± 0.858	3.447 *	0.031	5.190 ***
calories of food	2.07 ± 0.960	5.207 **	1.237	1.006
avoiding salt and sugar	1.72 ± 0.780	1.721	1.855	2.354 *
appropriate portions of food	2.64 ± 0.836	1.745	0.450	1.459
sanitation	3.13 ± 0.800	0.375	0.368	1.909

* *p* < 0.05, ** *p* < 0.01 *** *p* < 0.001. BMI categorized as 1 = under-weight, 2 = normal weight, 3 = over weight, 4 = obese.

**Table 3:** Characteristics of eating out.

Characteristics	N (Valid%)	BMI	Place of Residence	Gender
			χ^2^	
*With whom do you dine out?*				
Alone	26 (8.3%)			
Friends	215 (68.5%)	14.147	29.206 **	9.918 *
Family	68 (21.7%)			
Co-workers	1 (0.3%)			
Someone-else than above	4 (1.3%)			
Missing	13			
*What food do you prefer when dining out*				
Korean	159 (50.2%)			
Chinese	17 (5.4%)			
Japanese	47 (14.8%)	7.776	12.951	5.931
Western	57 (18%)			
Others	37 (11.7%)			
Missing				
*Budget for dining out (per person)*				
Less than 5000 won	4 (1.2%)			
5000~6999 won	68 (21.2%)			
7000~8999 won	109 (34%)			
9000~10,999 won	55 (17.1%)	16.299	17.957	10.117
11,000~12,999 won	55 (17.1%)			
13,000~14,999 won	9 (2.8%)			
15,000 won and above	21 (6.5%)			
Missing	6			

* *p* < 0.05, ** *p* < 0.01.

**Table 4:** Characteristics of RTE food purchase behaviors.

Characteristics	N (Valid%)	BMI	Type of Residence	Gender
*What is the purpose of purchasing RTE food*				
Breakfast	31 (9.7%)	24.459	13.654	3.045
Lunch	123 (38.6%)			
Dinner	73 (22.9%)			
Snack between meals	64 (20.1%)			
Night snack	28 (8.8%)			
missing	8			
*What is your ethnic preference for RTE food*				
Korean	166 (52.7%)	16.176	8.932	10.261 *
Chinese	14 (4.4%)			
Japanese	25 (7.9%)			
Western	50 (15.9%)			
others	60 (19%)			
missing	12			
*What is your budget for RTE food*				
Less than 4000 won	101 (31.5%)	16.071	16.433	10.720
4000~4999 won	124 (38.6%)			
5000~5999 won	75 (23.4%)			
6000~6999 won	12 (3.7%)			
7000~7999 won	8 (2.5%)			
8000 won and above	1 (0.3%)			
missing	6			
*What do you consider when purchasing RTE food* ※				
Price	164 (26.8%)	-	-	-
Taste	265 (43.3%)			
Brand	19 (3.1%)			
Portion	111 (18.1%)			
Nutritional value	31 (5.1%)			
Origins of ingredients	7 (1.1%)			
Low calorie	15 (2.5%)			
*Most considerable nutrients for RTE food?* ※				
Protein	75 (18.4%)	-	-	-
Carbohydrates	96 (23.5%)			
Fat	44 (10.8%)			
Cholesterol	36 (8.8%)			
Calorie	157 (38.5%)			

※ multiple answers. * *p* < 0.05.

## Data Availability

Not applicable.
